# Decoding Microglial Polarization and Metabolic Reprogramming in Neurodegenerative Diseases: Implications for Disease Progression and Therapy

**DOI:** 10.14336/AD.2024.1629

**Published:** 2026-02-19

**Authors:** Ran Gao, Ya Gao, Wenting Su, Renxi Wang

**Affiliations:** ^1^Beijing Institute of Brain Disorders, Laboratory of Brain Disorders, Ministry of Science and Technology, Collaborative Innovation Center for Brain Disorders, Capital Medical University, Beijing 100069, China.; ^2^Laboratory for Clinical Medicine, Capital Medical University, Beijing, China.; ^3^Department of Cardiology, The Second Medical Centre, Chinese PLA General Hospital, Beijing, 100853, China.

**Keywords:** microglia, microglial polarization, inflammation, metabolic reprogramming, neurodegenerative diseases

## Abstract

As the resident macrophages of the brain, microglia are crucial immune cells specific to the central nervous system (CNS). They constantly surveil their surroundings and trigger immunological reactions, playing a key role in various neurodegenerative diseases (ND). As illnesses progress, microglia exhibit multiple phenotypes. Traditionally, microglia have been classified into two main phenotypes upon activation: the pro-inflammatory M1 polarization and the anti-inflammatory M2 polarization. However, this classification is now considered overly simplistic, as it is unable to fully convey the intricacy and diversity of the inflammatory response. Immune regulatory factors, such as chemokines secreted by microglia, are essential for modulating brain development, maintaining the neural milieu, and orchestrating responses to injury, along with the subsequent repair processes. However, in recent years, the significance of metabolic reprogramming in both physiological microglial activity and ND has also become increasingly recognized. Upon activation—triggered by brain injury, infection, or ND—microglia typically modify their metabolic processes by transitioning from oxidative phosphorylation (OXPHOS) phosphorylation to glycolysis. This shift facilitates rapid energy production but may also enhance pro-inflammatory responses. This review seeks to summarize metabolic reprogramming and polarization in the function of microglia and their involvement in ND.

## INTRODUCTION

Within the CNS, microglia serve as the principal immune cells, consisting of roughly 10-15% of brain cells [1]. They are vital for sustaining neural equilibrium and facilitating immune monitoring. Originating from the yolk sac during embryonic development, microglia migrate to the CNS, where they are essential in early developmental processes [2]. Resident microglia in the brain act as the primary defense against disturbances in physiological balance. These cells uniquely oversee the microenvironment and dynamically survey the entire brain by continuously extending and withdrawing their branched projections [3]. In neurodegenerative diseases, microglia undertake multiple immune roles, such as phagocytosis, cytokine secretion, and the production of reactive oxygen species (ROS), altering their phenotype in response to shifts in the surrounding environment [3-5]. These different phenotypes help the immune system in the nervous system to control and maintain tissue homeostasis [6]. In the context of chronic neuro-inflammation, microglial metabolic profile also undergoes significant changes.

ND, including Parkinson's disease (PD), Alzheimer's disease (AD), and amyotrophic lateral sclerosis (ALS), are chronic conditions that lead to neuronal degeneration in the peripheral and central nervous systems [7,8]. The global socio-economic impact of these diseases is increasing rapidly. In ND, microglia become overactivated and release large amounts of inflammatory mediators, creating an inflammatory microenvironment [9,10]. Upon activation, microglia change their phenotype and continuously assess and clear brain tissue metabolites and debris. They actively participate in synaptic remodeling, neuronal damage, and repair, all of which have high energy demands [11,12]. Metabolic reprogramming refers to the method by which cells adapt their metabolic profiles to meet the energy and biomolecular requirements of their surrounding microenvironment. Recent research has emphasized the critical function of microglial metabolic reprogramming in immune metabolism and neurodegenerative disorders, particularly in relation to amino acid, glucose, and fatty acid metabolism.

Like other cells, microglia primarily use glucose as an energy source. Their functional changes are closely linked to the advancement of ND, but whether microglial activity exacerbates, or limits neurodegeneration remains a subject of ongoing investigation. Researchers consider neuroinflammation and its associated metabolic changes to be key factors in the beginning and development of these conditions. Recent studies have underscored the significance of metabolic reprogramming in microglia for regulating their polarization and functionality, particularly in relation to neuroinflammatory responses and neurodegeneration [13-15]. However, the specific changes and metabolic pathways involved in microglial glucose metabolism during ND, as well as their mechanisms of interaction with neuroinflammation remain incompletely understood.

This article offers a thorough and up-to-date review of the evolving roles of microglial activity and key polarization processes in the development of ND. Finally, we will address the existing challenges and potential future avenues for exploring the complex interaction between microglial metabolism and polarization within the framework of ND.

## MATERIAL AND METHODS

### Search strategy

**Figure 1.**
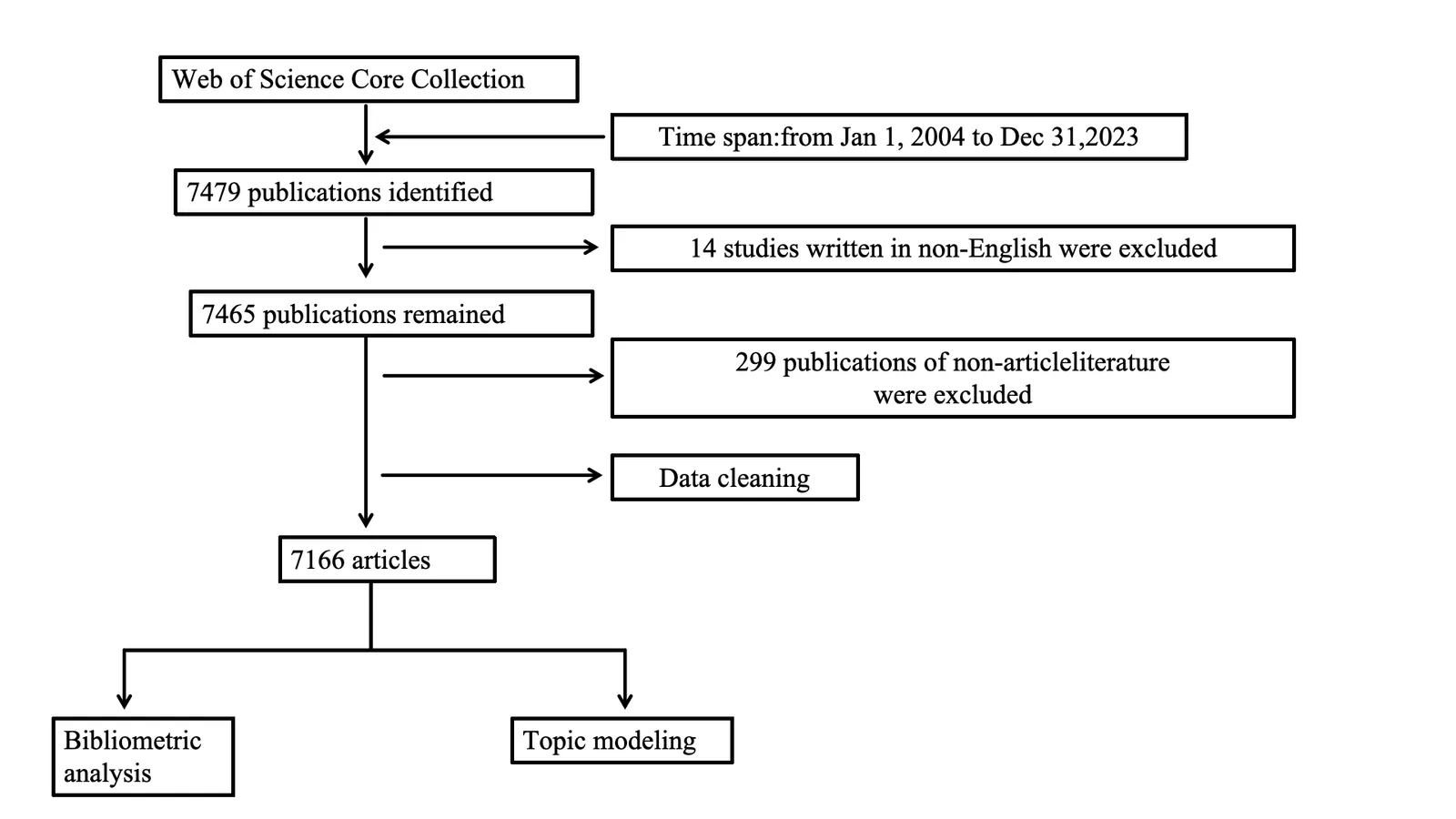
Literature search and selection.

As in previous studies [16], the analysis began with a preprocessing step to clean and prepare the downloaded data for subsequent analysis. We removed articles without summaries to ensure a comprehensive, complete data set. We sourced the data for this analysis from the Web of Science Core Collection (WOSCC), which provides the most comprehensive bibliographic data available. The time frame of the search spans from 2004 to 2023, with the search restricted to English-language reviews and articles. The search was completed on November 24, 2024, as outlined in Figure 1.

### Data collection and methods

As described in previous studies [17,18], VOSviewer (1.6.18, Leiden University) and CiteSpace (5.8) were used to conduct bibliometric analysis, focusing on temporal trends and keyword visualization. CiteSpace analyzed and visualized keyword bursts, with nodes representing keywords and their associated timelines. VOSviewer performed keyword clustering. After importing the downloaded data, we selected options such as "co-authorship," "co-occurrence," and "author" to achieve our research goals and set a threshold of 190-200 documents to filter the data.

In the visual output from VOSviewer, node size reflects the frequency, quantity, and citation count of keywords, while link thickness indicates the strength of relationships such as collaboration or co-occurrence. VOSviewer automatically clusters data based on keyword frequency and assigns distinct colors to each cluster.

CiteSpace was used to analyze and visualize keyword bursts; nodes represent keywords and their associated timelines. CiteSpace is similar to VOSviewer in terms of data processing. When using CiteSpace for time trend analysis, the source selects all items, including article, and other keywords selected for nodes. By setting a time slice for one year to check the changing trend of some key terms or concepts in different periods, retrospective analysis, and summary of the literature in microglia and neurodegenerative diseases in the past 20 years, the development vein and emerging hot topics in the research field can be revealed and predict its research frontier. Figure 1 displays a flow diagram of this search method and selection procedure.

### Microglial Functions

Microglia are the brain's local macrophages, cultivated during the development of the embryo and maintained through self-renewal without external input [19,20]. Under physiological conditions, microglia play a crucial role in preserving brain balance by monitoring the brain's microenvironment. Neurons in the CNS rely on a stable microenvironment for survival, and their maintenance largely depends on innate immune cells, specifically microglia [1,21-23]. During development, microglia regulate brain function by modulating programmed cell death and synaptic maturation [2]. As the main immune cells in the brain, microglia capture pathogens and remove cellular debris to restore CNS homeostasis.

Several investigations have shown that microglia serve as neuroprotective agents in various mechanisms. For example, in a neural injury model, researchers studied the contribution of microglia to nerve injury repair by isolating nuclei associated with cell types to label microglia for polarization analysis and functional assessment. These processes revealed that microglia contribute to nerve damage repair by promoting neurogenesis, synaptic pruning, and inhibiting harmful inflammation through the secretion of anti-inflammatory factors [24-27].

However, during disease progression, microglia can adopt dysfunctional phenotypes and produce cytotoxic effects, which have been shown to negatively impact conditions such as ND [28,29]. For example, researchers linked microglial polarization to changes seen in severe neurodegenerative conditions, including AD, PD, HD, MS, and ALS [30-32]. Some theories suggest that microglia-driven neuroinflammation and metabolic changes could contribute to the neurodegeneration, though the precise underlying processes remain unclear.

### Polarization process

Microglia undergo complex, long-term changes during polarization in response to neuroinflammatory conditions. A defining feature of microglia is their ability to undergo transformation in response to pathology within the CNS. Researchers now understand this transformation as a continuum, encompassing intermediate stages that involved alterations in gene expression, migration, metabolism, sensory functions, secretion, phagocytosis, and cell death [33,34].

Neuroinflammation is characterized by microglial polarization, which refers to the process by which microglia, the resident immune cells of the CNS, adopt distinct functional states in response to various stimuli, such as injury or environmental factors [35-37]. These polarized states are commonly categorized into the M1 pro-inflammatory or M2 anti-inflammatory phenotypes to respond to the disturbances in the microenvironment [38,39].

M1 polarization usually refers to microglial activation of surface receptors such as TLRs upon exposure to bacteria-derived products or infection-related signals, as shown in Table 1. Pro-inflammatory molecules produced by pathogens or injured cells stimulate resting microglia to produce pro-inflammatory substances like IL-1β, TNF-α, and proteases that affect neurons and surrounding immune cells during neuroinflammation [40-42]. By promoting local inflammatory responses, microglia effectively remove foreign pathogens or damaged cells and initiate nerve repair processes. Additionally, M1 microglia are continuously activated, and an excessive pro-inflammatory response causes persistent inflammation inside the neurological system, promoting neurodegeneration and accelerating disease progression.

Conversely, mediators like IL-4, IL-10, and IL-13 are mainly secreted by M2 microglia. These stimulate neuroprotective responses in microglia, prompting the secretion of cytokines such as FIZZ1, IL-4, arginase 1, Ym1, CD206. These factors may be involved in neuroprotection and tissue repair; researchers demonstrated that IL-4, for instance, suppressed the secretion of pro-inflammatory factors [43].

**Figure 2.**
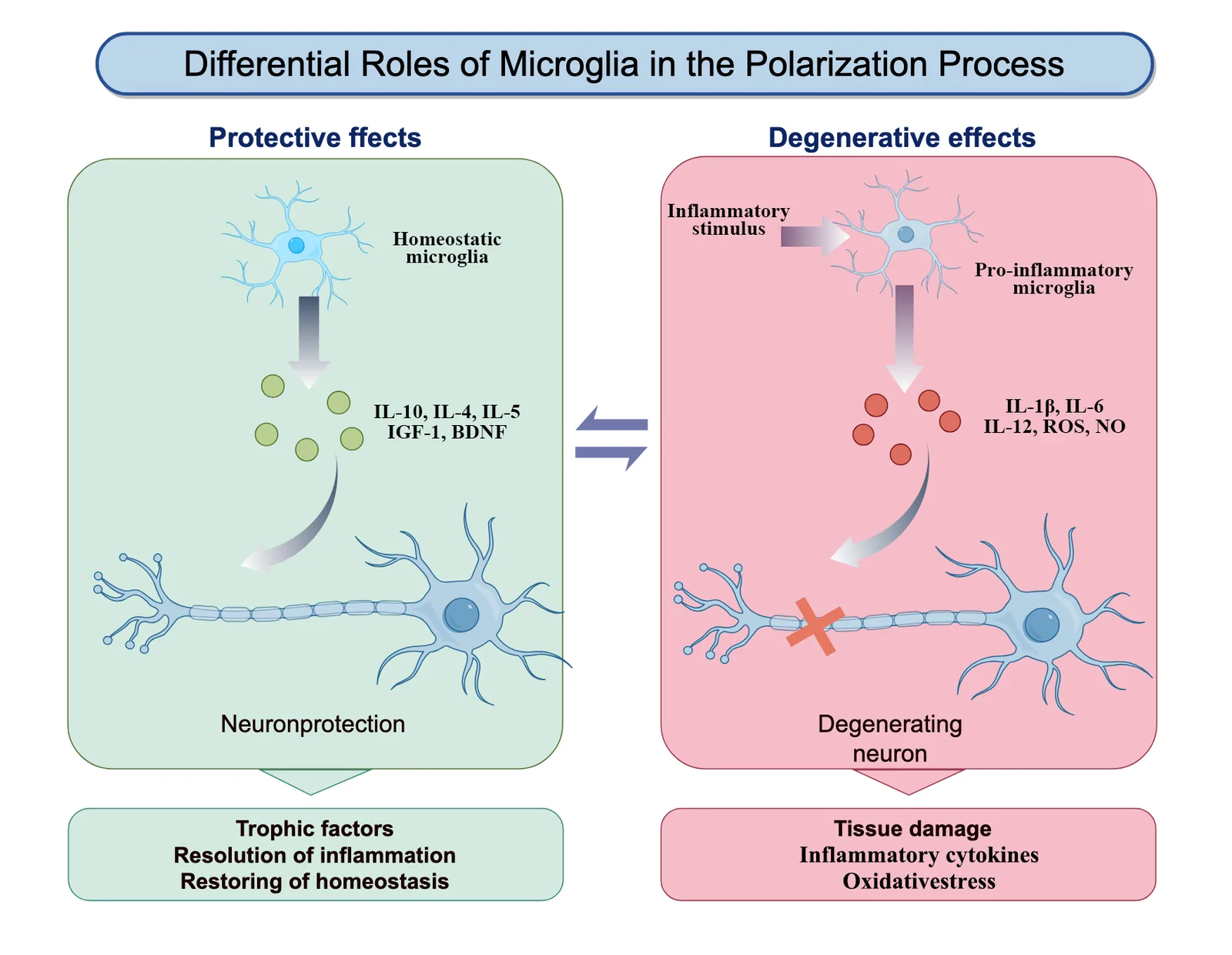
**The regulatory role of M1/M2 microglial polarization in CNS**. Microglia in their resting state secrete IL-4, IL-13, or IL-10, inducing M2 polarization while suppressing M1 activity. By discharging neurotrophic substances and anti-inflammatory cytokines, the M2 phenotype offers neuroprotection and mitigates neuronal damage. By contrast, under stimulation by LPS and IFN-γ, microglia adopt an M1 phenotype. This shift leads to the generation of pro-inflammatory mediators including IL-1β, IL-6, CCL2, NO, and ROS. These factors collectively establish a toxic inflammatory milieu, impairing neuronal function and ultimately precipitating neuronal death.

Recently, studies demonstrated that M2-type microglia play a harmful role in some cases. For example, the immune response may be excessively suppressed in some ND, leading to a reduced ability to clear pathological proteins. As a result, these pathological changes are not effectively eliminated, which in turn exacerbates disease progression [43-45]. Therefore, understanding the balance between M1 and M2 microglia and their dual roles in ND is important for the clinical development of more precise treatment strategies (Fig. 2).

However, recent research has shown that the traditional M1/M2 dichotomy is overly simplistic and unable to capture the complexity of microglia [46]. Microglial phenotypes are more intricate than what is represented in *in vitro* models, and they are highly plastic and diverse. Some microglia neither distinctly express markers of the M1 or M2 phenotypes nor display bio-homogeneity when expressing similar polarizing markers [47,48]. Microglia exhibit distinct phenotypes in homeostasis and can shift into different activation states in response to stimulation [49]. These phenotypic states are not static but exist along a continuum, reflecting the dynamic processes within microglia [48].

**Table 1:** Surface reactive receptors of microglia: functional characteristics and associated pathways.

Receptor	Recognized structure/ligand	Associated pathway	Functional characteristics	Ref.
**Toll-like receptors (TLRs)**	Pathogen-associated molecular patterns (e.g., LPS, dsRNA, CpG DNA)	NF-κB, MAPK	Detects microbial pathogens, initiating pro-inflammatory cascades and inducing cytokine release	[59]
**NOD-like receptors (NLRs)**	DAMPs (e.g., ATP, uric acid)	NF-κB, Caspase-1	Senses cellular injury, activates inflammasome complexes, and mediates the regulates of IL-18 and IL-1β	[60,61]
**RAGE**	HMGB1, S100 proteins	NF-κB, MAPK	Drives chronic inflammation, promoting the generation of ROS and the release of inflammatory substances	[62,63]
**CD36**	Oxidized low-density lipoprotein (oxLDL)	Src, MAPK, PI3K-Akt	Recognizes modified lipids, participates in the removal of apoptotic cells, and regulates metabolic homeostasis	[64,65]
**Dectin-1**	β-glucan (component of fungal cell wall)	Syk, CARD9, NF-κB	Recognizes fungal infection, induces pro-inflammatory response and antimicrobial immunity	[66,67]
**P2X7**	ATP	MAPK, NF-κB, Caspase-1	Induces inflammasome activation via ATP signaling, promotes IL-1β release	[68]
**TREM2**	Phospholipids, apoptotic cells	PI3K-Akt, NF-κB	Promotes microglial proliferation, phagocytosis, and anti-inflammatory response	[69]
**Fc Receptors**	Antibody-antigen complexes	Src, Syk, NF-κB	Mediates antibody-dependent phagocytosis, regulates adaptive immunity	[70]
**CR3 (Complement receptor 3)**	C3b, iC3b	PI3K-Akt, NF-κB	Recognizes complement-tagged pathogens, promotes phagocytosis and inflammatory response	[71,72]
**CX3CR1**	CX3CL1 (ligand from neurons)	PI3K-Akt, ERK	Regulates neuron-microglia interaction, primarily exhibits anti-inflammatory effects	[73]
**Scavenger Receptors**	Modified proteins, apoptotic cell debris, oxidized lipids	MAPK, NF-κB	Clears cellular debris and pathogens, regulates tissue homeostasis and inflammation	[59,74]

Likewise, microglia activation might show up as either pro- or anti-inflammatory; yet this transformation is a transitional, constant change impacted by elements including injury and inflammation [50]. The phenotype of microglia is not only shaped by the external environment but is also closely linked to their intrinsic characteristics and the maturation process of the CNS, which endows microglia with a high degree of phenotypic plasticity and diversity [48]. These phenotypes fall on a dynamic continuum and exhibit varying immune characteristics based on the severity and phase of the illness [51].

Applying single-cell technologies has revealed significant differences in microglial response patterns, particularly their more complex behaviors under different stimulus. Some studies have even shown that individual microglial cells express both M1 and M2 markers, as well as a range of M2 phenotypic subsets, including M2a, M2b, and M2c, each possessing distinct physical characteristics and biological features [52]. As a result, these phenotypic states have distinct physiological and pathological effects.

The functional state of microglia varies greatly in different environments, and this phenotypic transformation, intricately linked to their function, plays a significant role in the development of ND [53]. In AD, microglia typically display a pro-inflammatory M1 phenotype, particularly around Aβ plaques. Recent studies suggest that in the AD environment, microglia are prone to polarization toward the damaging M1 phenotype [54,55]. Additionally, new research employing clinical techniques like spatial transcriptomics and single-cell RNA sequencing provides deeper insights into microglia's gene expression profiles in different environments [56,57].

Although the classical categorization of microglia into M1 and M2 is overly simplistic, it remains a useful starting point for understanding the complex microglial phenotypes observed in neurodegenerative diseases. This understanding could enhance our comprehension of microglial function throughout disease progression and may help guide the creation of new treatment strategies. Multiple investigations have demonstrated that controlling microglial polarization is crucial for alleviating central nervous system diseases [35,58].

### Microglial metabolic reprogramming

Metabolic reprogramming, a concept initially introduced by Warburg to describe the unique energy metabolism of tumor cells, has also been observed in microglia [75]. These immune cells of the CNS exhibit similar shifts in metabolic pathways, closely tied to their activation states [76]. The energy demands of microglia primarily arise from the breakdown and metabolism of various nutrients, including glucose, lipids, and proteins [15,77]. Molecules associated with glucose metabolism, such as glucose transporters (GLUTs), are pivotal in the metabolic reprogramming of neurodegenerative diseases mediated by microglia. Table 2 summarizes the key molecules that regulate changes in microglial glucose metabolism under inflammatory conditions, detailing their respective functions and related findings.

**Table 2:** Key molecules involved in glucose metabolism changes in microglia and their correlation.

Molecule	Function	Effects under inflammation	Reference
**GLUTs**	Facilitate glucose uptake, regulate microglial metabolism	Inflammatory stimuli increase GLUT1 expression, enhancing glycolysis and lactate release; GLUT1 inhibitors reduce the release of inflammatory mediators (IL-1β, TNFα, and IL-6 etc)	[96,97]
**Hexokinase II (HK2)**	Catalyzes the initial step of glucose metabolism, regulates glycolysis and mitochondrial activity	HK2 deficiency promotes M1 polarization, enhancing phagocytosis and inflammatory responses	[98,99]
**Phosphofructokinase 1 (PFK1)**	Regulates the rate of glycolysis, modulated by the PFKFB3 isoform	PFKFB3 is upregulated under pro-inflammatory conditions, promoting glycolysis, restoring oxidative metabolism, and reducing inflammation	[100]
**Monocarboxylate Transporters (MCTs)**	Transport pyruvate, lactate, and other monocarboxylates	Expression of MCT1, MCT2, and MCT4 is elevated upon M1 polarization; MCT1 inhibition reduces glycolysis and inflammatory cytokine expression	[101-103]
**Pyruvate Kinase M2 (PKM2) and Pyruvate Dehydrogenase (PDH)**	End enzymes of glycolysis and acetyl-CoA production	PKM2 levels are elevated in AD samples, influencing M1 polarization; PDH is linked to the balance of oxidative metabolism	[104-106]
**Lactate Dehydrogenase (LDH)**	Facilitates the conversion of pyruvate to lactate, maintaining glycolytic flux	LDH is highly expressed in microglia, and lactate production is enhanced during pro-inflammatory responses	[107,108]

Depending on the energy sources it uses, each type of cell displays distinct metabolic traits under physiological conditions. However, alterations in cellular activity within the surrounding microenvironment can induce transformations in these metabolic profiles. For instance, in response to different inflammatory signals, microglia can alter their intracellular metabolic pathways, as illustrated in Table 3. The investigation of these molecules offers valuable insights into the intricate relationship between microglial metabolic reprogramming and neurodegenerative diseases while also providing a path for the creation of alternative treatments.

Increased glycolytic flux, accumulation of specific TCA cycle metabolites, decreased mitochondrial respiration, and improved transcriptional control of HIF-1α and mTOR are characteristics of metabolic reprogramming [78,79]. Recent studies show that iron deposition in microglia induces inflammation and glycolytic phenotypes, increases ferritin expression, and raises TNF-α and IL-6 secretion [45]. It also impairs glycolysis and microglial function, reducing phagocytosis and chemotaxis [80,81]. These changes modulate the microglial immune functions, such as cytokine synthesis, ultimately contributing to ND [82].

When faced with metabolic stressors, cultured microglia can activate alternative glycolytic pathways or expand mitochondrial respiratory capacity, positioning them as universal sensors of physiological changes in the CNS [83,84]. These cells combine multiple cellular signals and conduct extensive phenotypic changes to adjust to inflammatory events. However, direct data on the metabolism of microglia within brain tissue remains limited [85-87]. Recent studies using fluorescent lifetime imaging of acute brain sections, which represent the backbone paradigm for researching the CNS from circuitry to nanoscale occurrences, have revealed time-dependent phenotypic changes in microglia impacted by intricate extracellular ATP dynamics via P2Y12R and CX3CR1 signaling [88]. *In vitro*, the signal persisted in mouse brain slices for several hours, and the results showed that while microglia are more glycolytic than other cells in the neocortex, they exhibit significant metabolic flexibility and can participate in glutamyl amylolysis, enabling them to actively survey the brain parenchyma [88-90].

**Table 3:** Key metabolic pathways of microglia.

Target/pathway	Composition/activation mechanism	Function	Mechanism	Disease relevance	Inhibition/activation effect	Reference
**mTOR**	Composed of mTORC1 and mTORC2; activated by PAMPs (LPS) and DAMPs (ATP)	Regulates GLUTs, glycolytic enzymes, and transcription factors	Promotes glucose utilization, modulates inflammation, increases glycolysis	Associated with aging and neurodegenerative diseases	Inhibition reduces glycolysis, ROS, and pro-inflammatory factors	[109,110]
**HIF-1α**	Composed of HIF-α and HIF-β; upregulated under hypoxic conditions, downstream of mTOR	Controls metabolic reprogramming, promotes glycolysis, inhibits TCA cycle	Increases GLUT1, HK2 activity, reduces OXPHOS, stabilizes pro-inflammatory state	Linked to Aβ toxicity and neurodegeneration	Inhibition reduces pro-inflammatory cytokine expression, alleviates neurodamage	[111,112]
**PI3K-AKT-mTOR**	Composed of PI3K, AKT, mTOR; activated by LPS stimulation	Regulates glycolysis and pro-inflammatory gene transcription	Upregulates PDK1, enhances glucose uptake, promotes glycolysis, reduces OXPHOS	Promotes inflammation in neurodegenerative diseases	Inhibition decreases STAT3 phosphorylation, reduces inflammation	[113]
**AMPK**	Composed of AMPKα, AMPKβ, AMPKγ; activated during energy stress	Regulates OXPHOS, glucose and lipid metabolism, maintains metabolic balance	Increases GLUT1 and GLUT4 translocation, enhances glycolysis, supports anti-inflammatory state	Involved in metabolic dysregulation and chronic inflammation	Activation inhibits glycolysis, NLRP3, and pro-inflammatory gene expression	[114,115]

These findings imply that microglial functions are sustained by intricate metabolic networks within the brain, potentially involving the dynamic exchange of energy substrates between different cell types. At the same time, this phenomenon highlights the critical reliance of microglia on glycolysis and oxidative energy metabolism, with this metabolic balance closely tied to their activation states and shifts under various pathological or physiological conditions. This underscores the intimate link between microglia and metabolic reprogramming, offering insights into their adaptive response mechanisms and presenting a fresh perspective for understanding their role in ND.

Comparative transcriptomic analyses of energy metabolism-related genes in neurons, astrocytes, and mouse microglia reveal that microglia uniquely express a repertoire of genes essential for both glycolysis and oxidative phosphorylation [91-93]. This metabolic trait suggests that metabolic imbalance within microglia may be a key pathophysiological basis for a number of brain illnesses in addition to the crucial function that microglia play in preserving brain homeostasis.

Recently, the link between microglial metabolism and its functions, particularly in ND, has gained increased attention. The results from mouse models provide useful preliminary data but cannot fully replicate the complex metabolic and immune responses seen in human disease. Studies have shown that iPSC-derived microglia, brain organoids, and transplantation into humanized mice can simulate inflammation and metabolism *in vivo* [94,95]. However, despite the vast therapeutic potential of this area, our understanding of the metabolic alterations in microglia under disease conditions remains significantly limited.

Using hippocampal slice cultures, researchers found that the increase in glucose consumption and lactate secretion depended on microglial activity in inflammatory cortical tissue. This suggests that microglia not only regulate their own metabolism in inflammatory environments but may also influence other brain cells through the secretion of metabolites [85]. Future studies will aim to further elucidate the key interactions between microglial metabolism and their communication with other brain cells. This will help reveal how these dynamic metabolic processes influence the course of neuroinflammatory diseases and pave the way for novel approaches targeting microglial metabolism.

### Microglia and ND

**Figure 3.**
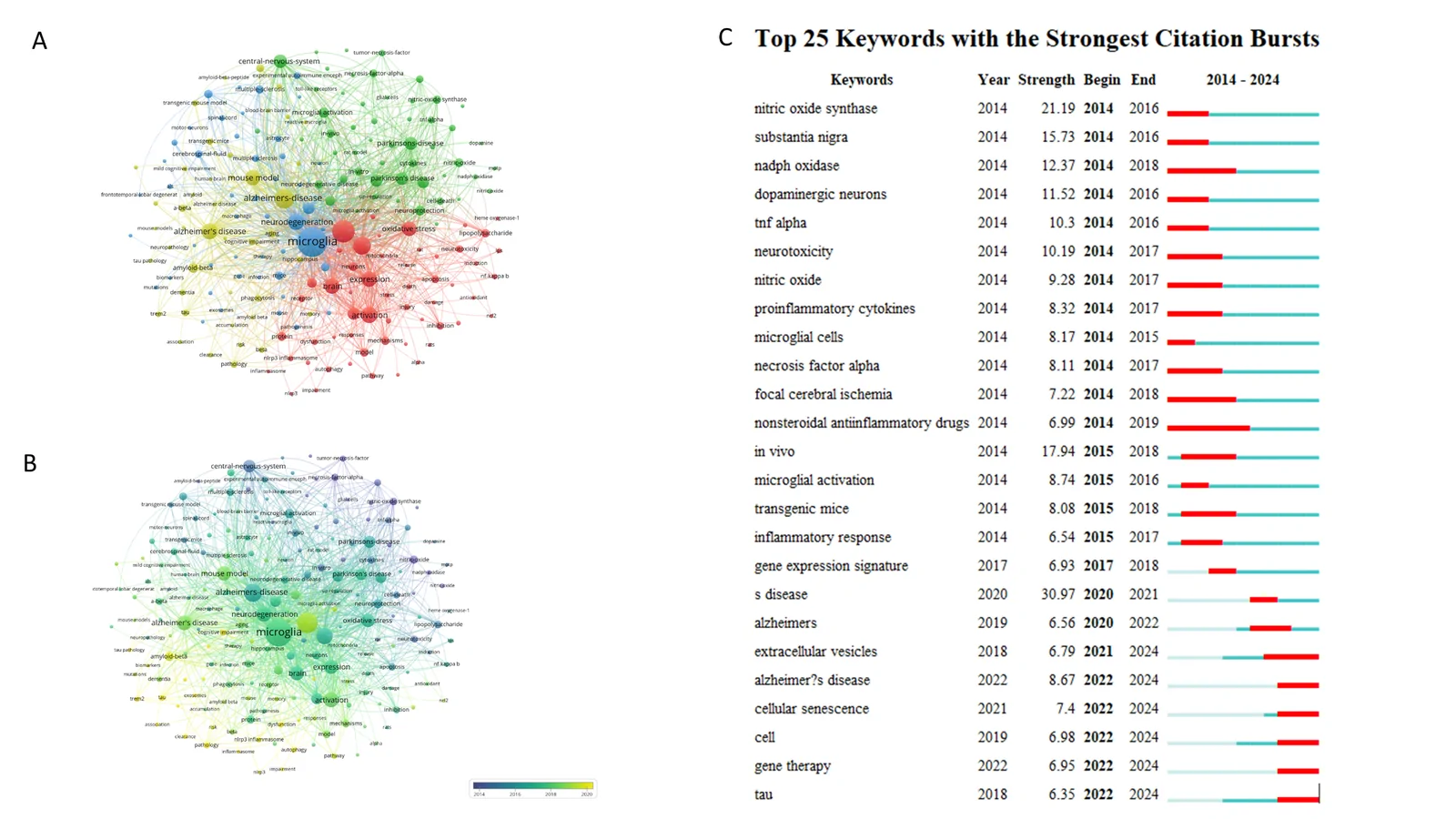
**The keyword distribution map provides a visual representation of research topics and their evolutionary trends, with each node representing a keyword**. (**A**) Clustering map of keywords: This map categorizes keywords based on co-occurrence, with different colors representing distinct clusters. These clusters highlight the connections and distinctions between various research topics, offering insight into the thematic structure of the field. (**B**) Temporal clustering map: This map is based on the average appearance time of keywords, where gradient colors represent the temporal distribution. Darker colors correspond to keywords with earlier average appearance times, while lighter colors indicate keywords that have appeared more recently. This visualization reveals the chronological development and trajectory of the research field. (**C**) Keyword burst map: Highlighting keywords with high-frequency occurrences within specific timeframes, this map identifies research hotspots and trends. It provides valuable references for understanding emerging focus areas and shifts in research priorities.

Using existing data, we conducted a comprehensive keyword analysis of research trends in microglia and neurodegenerative diseases from 2004 to 2023, based on the WOSCC. The keyword analysis reveals core research areas and emerging hotspots. From 7166 articles, we identified 197 keywords, 126 of which appeared more than 100 times. Figure 3A displays a network grouping keywords into color-coded clusters. Figure 3B shows an overlaid visualization map based on their temporal patterns. Figure 3C highlights the top 25 keywords with the greatest citation bursts, with red lines indicating the outbreak periods, including start and end years (Fig. 3).

We identified four clusters for keyword co-occurrence analysis (Fig. 3A). A red cluster primarily focuses on the pathological and physiological mechanisms of microglia and neurodegenerative diseases, with key terms such as "neurodegeneration," "neuroinflammation," "oxidative stress," and "activation." The strong correlation between "neuroinflammation" and "oxidative stress" highlights their continued importance as critical research areas, with "biomarkers" emerging prominently since 2018, indicating their status as a recent research hotspot. Green and blue clusters mainly encompass keywords related to specific ND, particularly PD, emphasizing their position as mainstream research topics. These clusters include terms like "neuroprotection," "apoptosis," and "cognition," underscoring their close association with microglial function. A yellow cluster focuses on research models and phenotype analysis, particularly in the context of cognitive decline, reflecting a core interest in neurodegenerative disease research.

**Figure 4.**
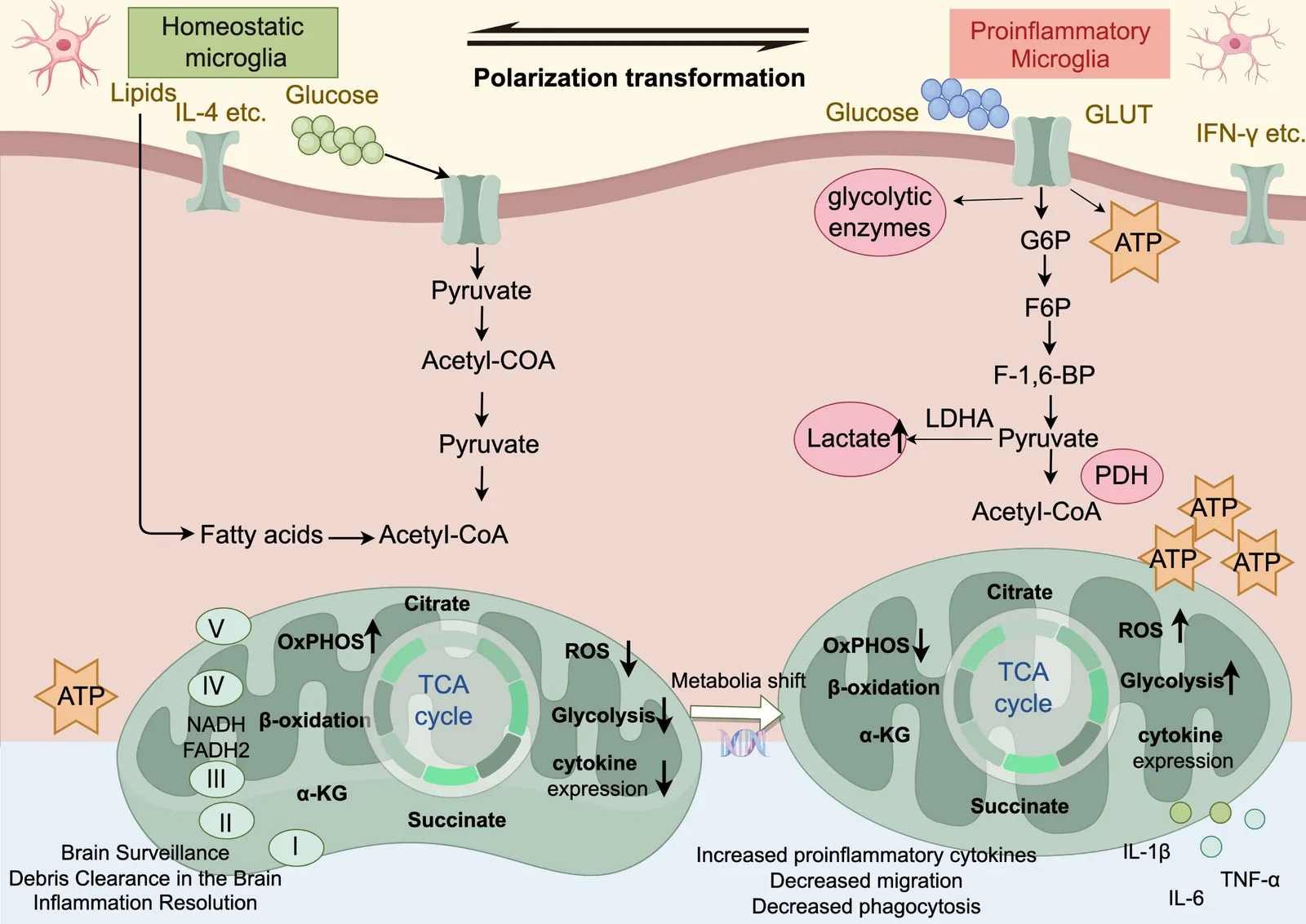
**Microglial polarization and metabolic reprogramming**. In a healthy state, microglia predominantly utilize OXPHOS to produce energy, supporting brain repair, enhancing phagocytosis, and releasing neurotrophic factors. During inflammation, they undergo a metabolic shift toward glycolysis, marked by increased glucose absorption, lactate generation, succinic acid accumulation, lipid synthesis, and upregulation of glycolytic enzymes. This metabolic reprogramming amplifies inflammatory responses while impairing phagocytic functions. The distinct phenotypes of microglia are closely associated with specific metabolic pathways, with activation inducing changes in mitochondrial dynamics and enabling transitions between glycolysis and OXPHOS to meet functional demands.

Figure 3B reveals a notable shift in research focus over time. Early studies centered on classical mechanisms such as inflammatory factors and oxidative stress in ND, but recent trends have moved toward exploring metabolic reprogramming, autophagy, and polarization signaling pathways, suggesting a transition towards more precise therapeutic approaches for neurodegenerative diseases. These findings align with the sudden emergence of keywords in Figure 3C, indicating a shift from classical mechanisms to precision medicine. The transition highlights the importance of understanding molecular mechanisms, particularly the polarization and metabolic reprogramming of microglia. The deeper exploration of these processes could provide critical insights for developing effective treatment plans for ND, emphasizing the potential of precision medicine in addressing these complex conditions (Fig 4).

### Polarization and metabolic processes of microglia

Today, the effects of the microglial polarization process on the metabolism of cellular energy are well established. In the classically M1 type, metabolic reprogramming shifts toward glycolysis, accompanied by the generation of nitric oxide (NO) and citrulline [116,117]. This metabolic switch enhances glucose uptake, lactate production, and PPP activation while reducing the consumption of mitochondrial oxygen.

In microglia, the common inflammatory stimulus like LPS can quickly induce aerobic glycolysis [118,119]. Recent findings show that the extracellular acidification rate (ECAR), a gauge of glycolysis, significantly increases within 150 minutes of LPS treatment [116], along with a significant increase in the expression levels of important glycolytic enzymes, including GLUT1 and HK2 [120]. These results underscore the ability of LPS to rapidly trigger glycolytic reprogramming in microglia.

Another characteristic of M1 is the generation of NOS, with NO produced via the oxidation of L-arginine, utilizing electrons provided by NADPH to power nitric oxide synthase (NOS), specifically inducible NOS (iNOS). When present in high amounts, NO reversibly impedes mitochondrial respiration by competing with oxygen for binding sites on cytochrome c oxidase. Consequently, as mitochondrial respiration diminishes, superoxide radicals (O_2_^-^) are produced, which are then enzymatically transformed by superoxide dismutase 3 (SOD3) into hydrogen peroxide (H_2_O_2_). Hydrogen peroxide diffuses into the cytoplasm, where prolonged NO production can lead to its reaction with O_2_^-^, forming peroxynitrite (ONOO^-^), an agent that irreversibly inhibits the electron transport chain [97,121,122]. In addition, NO suppresses the action of pyruvate dehydrogenase, a critical enzyme accountable for catalyzing the transition of pyruvate into acetyl-CoA [117,123].

Monitoring microglial polarization under these conditions suggests that while glycolysis increases, OXPHOS may still play a vital part in sustaining cellular energy metabolism. This highlights the significant impact of the polarization process on cellular energy regulation. Different polarization states may control energy supply and cellular function through distinct metabolic pathways. This metabolic regulation not only influences microglial polarization but also plays an important part in the initiation and advancement of neuroinflammation and ND.

We are only now starting to understand how energy metabolism influences microglial inflammation. One study shows that the glycolysis rate, independent of glucose levels, influences the formation of inflammasomes such as NLRP1 and NLRP3 [124], suggesting that the metabolic state of cells may partially regulate their inflammatory response. For example, the researchers restricted dietary energy through daily calorie reduction or intermittent fasting to examines the effects on the role of microglia in illness models. They found that microglia possess the capability to metabolize ketone bodies, such as acetic acid and β-hydroxybutyric acid, produced during ketogenic or fasting diets, and react to such metabolites as signaling molecules [125,126].

β-Hydroxybutyric acid triggers GRP109A receptors and prevents histone deacetylase in microglia, thereby suppressing their activation following brain injury and promoting their transformation into a neuroprotective phenotype [4,127]. This mechanism suggests that metabolites are essential for regulating microglia's inflammatory response and driving their transformation into protective phenotypes. In addition, studies used LPS as an inflammatory stimulus and the glycolytic inhibitor 2-deoxy-D-glucose to simulate the reduction in glycolytic flux induced by caloric restriction, suppressing the production of TNF-α and IL-6 via the nuclear factor kappa B (NF-κB) signaling pathway [97]. This discovery emphasizes the critical function of energy metabolism in regulating microglia's inflammatory response.

The precise regulation and disruption of energy metabolism may serve as a fundamental patho-physiological mechanism underlying various brain diseases [93,128,129]. In the context of ND, growing evidence suggests a strong relationship between the energy metabolism of microglia and their polarization state. As the metabolic pathways of microglia become better understood, future research is likely to uncover additional molecular mechanisms that regulate these processes, offering new therapeutic targets for interventions in neuroinflammation and ND (Fig. 4).

### Polarization of microglia and changes in energy metabolism programming in ND

ND are widespread disorders that primarily impact the CNS, and they are distinguished by gradual structural changes, neuronal degeneration, loss of function, and eventual cell death [3]. At present, there are no definitive cures for these illnesses, and the clinical efficacy of available drugs is either limited or difficult to detect. Typical ND include AD, HD, PD, multiple sclerosis (MS), and ALS. These conditions exhibit several shared subcellular pathological characteristics, such as aberrant protein accumulation, malfunctioning protein degradation mechanisms, disrupted axonal circulation, and pervasive bioenergetic and mitochondrial impairments [130-132]. Considerable evidence highlights the importance of microglial polarization within the brain in the beginning and course of ND. Similarly, disturbances in energy metabolism, particularly mitochondrial dysfunction, and glucose hypometabolism, have emerged as crucial early hallmarks of ND [133,134].

In ND, the interaction between microglial polarization and metabolic changes is particularly crucial. According to our investigation and research, microglia contribute to ND not only through immune responses but also by regulating the brain environment through their metabolic characteristics. The following section summarizes the changes in microglial polarization and metabolic reprogramming observed in major neurodegenerative diseases, offering insights into their potential roles in disease mechanisms and progression.

**Figure 5.**
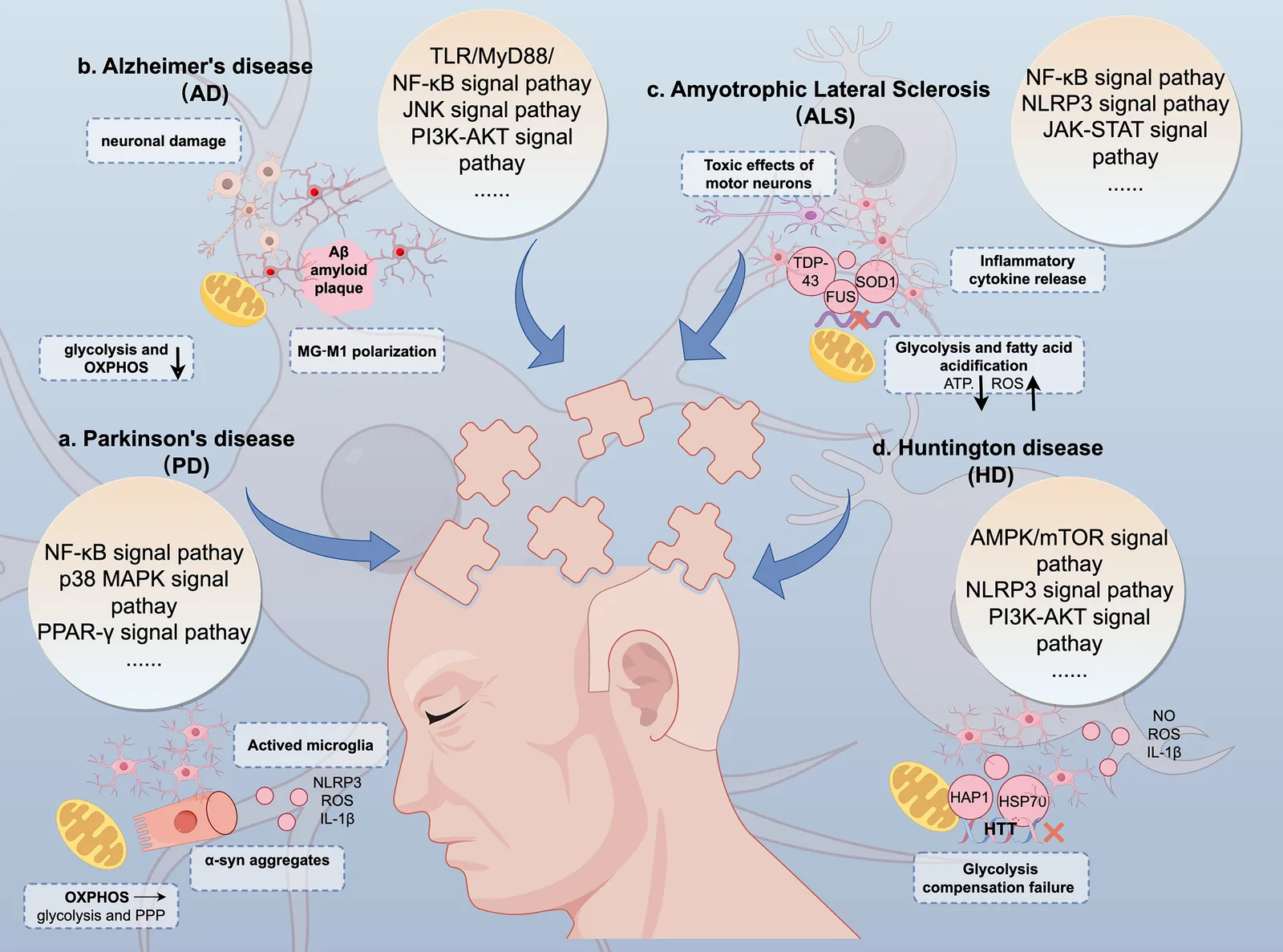
**The role of microglial polarization and metabolic reprogramming in ND**. An extensive synopsis of the functional alterations in microglial polarization and metabolic processes observed in ND such as AD, PD, ALS, and HD.

### AD

AD accounts for 60-70% of dementia cases and is defined by its hallmark amyloid-beta (Aβ) plaques and hyperphosphorylated tau protein tangles [59,135,136]. These pathological features contribute to synaptic loss, neuronal death, and cognitive decline. Genome-wide association studies (GWAS) highlight microglia as central players in AD development, and numerous AD risk factors are mostly or exclusively produced by these cells [137,138].

Neuroinflammation has a major impact on AD pathogenesis. Pro-inflammatory cytokines such as TNF-α, IL-1β, and IL-6 increase progressively from early disease stages, while others, including IL-18 and MCP-1, peak at specific points. Additionally, changes in TDP-43, progranulin, and TREM2, among other genes, impair defense mechanisms, intensify inflammation, and accelerate neurodegeneration [139-141].

A recent study analyzed 194,000 mononuclear microglial transcripts and epigenomes from 443 individuals with various AD pathologies [142]. The researchers identified 12 microglial transcriptional stages, including those related to AD-dysregulated homeostasis, inflammation, and lipid metabolism. These conditions show specific microglial roles linked to inflammation and lipid activity [142]. The study also examined the production of AD risk genes in conditions like microglial polarization. Unlike in mouse brains, microglial features were minimally enhanced over all conditions in this human dataset, suggesting species-specific differences in disease features [142]. Taken together, however, the data support the close link between microglial polarization and lipid metabolism in AD pathogenesis.

Emerging research highlights metabolic features of AD pathology, particularly in microglia [143]. In AD, M1 microglia undergo metabolic reprogramming, shifting from OXPHOS to aerobic glycolysis, a process referred to as the Warburg effect [144]. This shift increases glucose metabolism, promoting microglial functions like migration, proliferation, and phagocytosis in reaction to injury and other pathological stimuli [145]. Mitochondria are reprogrammed to meet the energy demands of these processes, but chronic inflammation produced by tau and Aβ disrupts the cellular TCA cycle, resulting in the buildup of metabolites such as citric acid, succinic acid, and cis-caprylic acid [146]. This exacerbates inflammation, inhibits OXPHOS, reduces ATP synthesis, and increases the production of saturated fatty acids [147]. These fatty acids can integrate into membrane phospholipids, making cells more vulnerable to inflammatory signals and lipid peroxidation, ultimately contributing to neuroinflammation and AD progression [147].

In AD patients, glycolytic activity of enzymes, including phosphofructokinase, aldolase, G6P isomerase, and LDH, is significantly reduced [148]. Additionally, mitochondrial defects, such as reduced activity of the pyruvate dehydrogenase complex and cytochrome oxidase, further indicate that metabolic abnormalities are a key feature of AD [148,149]. In APP/PS1 and 5xFAD mouse models, microglial metabolic changes are characterized by increased glycolysis and decreased oxidative phosphorylation [150,151]. These features support microglial immune and inflammatory responses in AD but may also exacerbate neurodegeneration.

While these models are valuable for studying AD mechanisms and drug screening, they have limitations in replicating the genetic diversity of clinical AD, as they only include APP and PS1 mutations [152]. To better simulate human AD, researchers have developed more complex double or multiple transgenic mouse models, incorporating mutations in tau, TREM2, APOE4, and other related genes [153,154]. For example, the APOE4 transgenic APP/PS1 mice showed earlier cognitive decline and were able to better mimic the early clinical manifestations of human AD patients [155,156].

### PD

PD is the most common movement disorder and the second most prevalent ND condition, trailing AD [157]. It is distinguished by the slow deterioration of dopaminergic neurons in the substantia nigra that project to the basal ganglia and are crucial for motor control [157].

Notably, the stimulation of glial cells, including microglia, and astrocytes, significantly contributes to PD pathogenesis. Research has shown that M1 polarization markers such as iNOS and COX2, along with pro-inflammatory enzymes and phagocytic markers like CD68, are elevated in PD patients [158]. PET scans of PD patients have shown extensive M1 polarization, with the level of activation correlating with dopaminergic terminal loss, especially in the early stages of the disease [159,160].

Microglia typically adopt the pro-inflammatory phenotype upon activation, and the release of aggregated alpha-synuclein from dead dopaminergic neurons can trigger this transformation. Overexpression of alpha-synuclein drives microglia into a reactive pre-inflammatory state, where inflammatory factors such as TNF-α, NO, and IL-1β further modulate neuro-inflammation in PD [158]. Additionally, signaling pathways like PPARγ are necessary for microglia to polarize. Activating PPARγ enhances the anti-inflammatory response and promotes the transition of microglia to the M2 type by inhibiting pro-inflammatory factors like STAT and NF-κB [76]. Anti-inflammatory cytokines, including TGF-β and IL-10 are released as a result, supporting tissue healing and lowering neuroinflammation. In summary, microglial polarization is a complex process influenced by various cytokines and signaling pathways, significantly impacting neuro-inflammation and ND.

According to recent research, the 6-hydroxydopamine (6-OHDA) rat model effectively replicates key characteristics of PD, including dopaminergic neuron loss and a classic Parkinsonian motor phenotype [161]. In this model, microglia rely on OXPHOS to produce ATP, which is essential for the proper functioning of neurons, particularly dopaminergic ones [162]. Glycolysis may also help neurons compensate for impaired OXPHOS, especially in energy-demanding neurons [163]. However, exposure to inflammatory triggers, including excessive alpha-synuclein PFF and neurotoxins, triggers metabolic reprogramming through the AKT-mTOR-HIF-1α pathway, shifting metabolism from OXPHOS to aerobic glycolysis [164,165], which results in M1 polarization and significant deficits in energy metabolism, phagocytosis, and immune function, ultimately leading to dopaminergic neuron loss.

The roles of OXPHOS and glycolysis in PD are complex and depend on the balance between the two pathways and their ability to adapt to pathological conditions. Research in both humans and PD animal models has shown increased G6PD expression, elevated PPP activity, and enhanced NADPH metabolism in microglia lead to excessive NOX2 activation [165]. This produces ROS, contributing to neuronal degeneration and dopaminergic neuron dysfunction. Additionally, the interaction between NOX2 and NO generates peroxynitrite, further exacerbating neuronal death [166].

Although the 6-OHDA model effectively replicates many of the features of PD, it still has limitations; its local lesions do not fully mimic the global brain degeneration of PD [167,168]. To overcome these challenges, researchers employ genetic modifications, induced iPSC technology, organoid models, and advanced small animal models to create more accurate and multidimensional PD research tools [147,169,170]. We expect these innovations to improve early diagnosis, mechanistic understanding, and drug development for PD.

### ALS

ALS, commonly mentioned as Lou Gehrig's disease, characterized by progressive neurodegeneration, stands as the most prevalent motor neuron disorder affecting adults. During this process, microglia undergo alterations in cell count, morphology, and the release of growth factors [171]. This leads to phenotypic changes, such as the alternating M2 phenotype or the classical M1 activation [172,173].

Different cytokines and signaling pathways regulate the activation of these phenotypes. In the M2 phenotype, IL-10 induces microglial inactivation and promotes their transformation into the M2 polarized state, while IL-4 supports the alternative M2 activation [174-176]. M2 microglia exert anti-inflammatory effects by clearing pro-inflammatory cytokines, regulating endothelial function, promoting tissue repair, and secreting elevated IGF-1 and IL-4 levels. By contrast, M1 microglia, typically activated by IFN-γ or LPS, secrete cytokines that promote inflammation, such as TNF-α and IL-1β, while inhibiting the release of nutritional factors, resulting in neuronal damage [177,178].

The transgenic mouse model expressing mutant human SOD1 (mSOD1) is the primary model of ALS. Studies have shown that microglia isolated from mSOD1 transgenic mice exhibit neuroprotective effects [179]. However, the neurotoxicity exhibited by microglia containing mSOD1 is dependent on NF-κB signaling and partly facilitated by IL-1β, further suggesting the pivotal role of microglia in ALS pathogenesis [180].

A previous study used human iPSC-derived microglia to examine the impact on microglial function, which closely resemble true human microglia [181]. This study provided a thorough phenotypic investigation of human C9orf72 variant iPSC microglia through RNA sequencing to identify a multitude of mechanisms linked to chemokines and immune cell activation in both C9orf72 hexanucleotide repeat expansion (HRE) mutant microglia and healthy controls. Most notably, after LPS initiation, pathways related to immune cell activation and cytokines were enriched [181]. This finding was consistent with the widespread M1 polarization clinically observed in patients with HRE, the most prevalent hereditary cause of ALS, which is linked to disease progression [182,183].

Disturbance of neuronal metabolic supply, particularly decreased glucose absorption and utilization, is thought to result from changed microglial types in ALS [184,185]. Bioenergetic flux analysis in the ALS mouse model SOD1-G93A revealed increased mitochondrial respiration and aerobic glycolysis via the activation of GLUTs, pyruvate dehydrogenase kinase 1, and MCT-1 in primary microglia [186-188]. Pro-inflammatory signals from microglia can disrupt mitochondrial dynamics, impair energy production, and increase oxidative stress, which contributes to the metabolic abnormality seen in ALS [189].

Additionally, microglia can respond quickly to inflammation by enhancing glycolysis to provide a rapid energy supply, which also leads to lactic acid accumulation. This accumulation can destabilize the intracellular environment, potentially triggering cell damage and creating a vicious cycle [190]. Although the SOD1-G93A mouse model is widely used and represents familial ALS, it does not fully capture the pathology of sporadic ALS, limiting the translation potential of research findings into treatments for this form of disease [191-193]. To address these challenges, clinical studies are incorporating other ALS models, such as the TDP-43 and C9orf72 mouse models, to obtain a more thorough comprehension of ALS pathology and processes [194,195].

These findings highlight the complex connection between neuroinflammation and metabolic diseases in ALS, underscoring the need for metabolic therapies to manage the disease. Ongoing investigation into ALS's metabolic pathways may result in new treatments that preserve neurons and improve patient states.

### HD

HD is a genetic ND marked by uncontrollable movements, cognitive impairment, and behavioral disturbances, all linked to the increase of trinucleotide (CAG) repeats in the Huntington gene [196]. In addition, a hallmark of HD pathology is reactive gliosis, primarily manifested as reactive fibroastrocytosis and M1 polarization in the striatum [197]. PET imaging studies have revealed notable microglial polarization in the HD brain's impacted areas, often detectable even before clinical symptoms emerge, with this activation becoming more pronounced in the disease's later stages [198-200].

The expression of mutant Huntington protein (mHTT) in neurons triggers M1 polarization through excitotoxicity or direct mHTT expression within microglia [199]. This polarization of microglia alters their functions and promotes neuroinflammation, a key factor in the progression of HD [201,202]. Researchers are exploring therapeutic approaches that aim to reduce mutant Huntington protein RNA (HtRNA) expression, alter microglial polarization, reduce inflammation, and provide neuroprotection.

Previous studies suggest that increased glycolysis in microglia can rapidly generate ATP, supporting migration, phagocytosis, and inflammatory responses in HD models [203]. This metabolic phenomenon helps clear mHTT aggregates and damaged cells, providing some neuroprotection [204,205].

In HD, primary microglia from adult R6/2 mice show impaired energy metabolism, leading to an accumulation of mHTT and enhanced glycolysis [206,207]. This, coupled with M1 microglial polarization, leads to the release of pro-inflammatory factors such as IL-1β [208]. The accumulation of mHTT may also impair oxidative phosphorylation by interacting with mitochondrial proteins, disrupting mitochondrial function, and producing excessive ROS [209,210]. These ROS further drive neuroinflammation and neuronal apoptosis. However, when oxidative phosphorylation is balanced, microglia can clear ROS and mitigate the toxic effects of mHTT on neurons.

PET imaging of HD patients shows increased microglial activity in regions like the striatum and frontal lobes correlates with areas of neurodegeneration [200,211,212]. Increased levels of pro-inflammatory cytokines, including IL-6 and IL-1β, have been detected in the cerebrospinal fluid and blood of HD patients, reflecting M1 polarization and metabolic reprogramming [200]. Animal models and clinical imaging and biomarkers support these findings. However, in animal models, microglial M1 polarization is often premature and rapid, whereas in patients, neuroinflammation generally progresses more gradually with disease progression [200,213]. Therefore, it is crucial to develop animal models that more closely mimic human HD pathology. This includes using transgenic models with moderate CAG repeats and applying single-cell transcriptomics to analyze microglial dynamics in both HD patients and animal models [214,215]. These approaches could help refine anti-inflammatory therapeutic strategies (Fig. 5).

### Current Therapeutic Strategies for ND

Currently, the treatment strategies for ND prioritize symptom alleviation and halting illness progression. While no cures exist, various drugs have been created to target the fundamental processes of ND to help manage clinical symptoms. Below are some of the existing treatment approaches for major neurodegenerative diseases (Table 4).

**Table 4:** Current treatment strategies for four major neurodegenerative diseases.

Disease	Therapeutic goal	Drugs/researchers	References
AD	Modulate neuroinflammation, enhance microglial phagocytosis of Aβ	NLRP3 inhibitors (OLT1177),N,N′diacetyl-p-phenylenediamine; Min Hee Park et al.	[10,216-218]
	**Activation of TREM2, altering microglial phenotypes, boosting Aβ clearance**	TREM2 antibodies (4D9, AL002c), bispecific antibodies, TREM2 plasmid; Pengzhen Wang et al.	[219,220]
	**Inhibit exosome synthesis to limit pathological tau propagation**	nSMase2 inhibitors, P2X7 receptor blockers; Asai H et al.	[82,121,220]
	**Promote microglial metabolic shifts to decrease amyloid burden**	NaR to stimulate OXPHOS; Rui-Yuan Pan et al.	[221-223]
	**Reprogram microglia, ensuring protective phenotypes for improved cognitive outcomes**	ROS-responsive polymeric micelles in AD models; Yifei Lu et al.	[224,225]
PD	Prevent M1 polarization, alleviating neuroinflammation	Naloxone, Xilei san; Liu B et al.	[226,227]
	**Phenotypic targeting of microglia for dopaminergic neuron protection**	PPAR-γ agonists (rosiglitazone), vitamin D; Pisanu A et al.	[228]
	**Microglial therapies under clinical investigation**	NLY01, AZD3241, MCC950; Jucaite A et al.	[229]
**ALS**	Reduce neuroinflammation, improve motor functions through microglial targeting	Riluzole, minocycline, stem cell therapies, SOD1 gene editing; Luo et al.	[230-232]
**HD**	Alleviate neuroinflammation, enhance motor performance by modulating microglia	Anti-inflammatory agents (minocycline, levetiracetam), HD-specific drugs (creatine, coenzyme Q10), HTT gene therapies	[233-235]

### Conclusion and Future Directions

In recent years, microglial polarization and neuroinflammation have become important contributors to the development of numerous ND. Microglia are essential to the development of ND through mechanisms including early activation, migration to injury sites, phagocytosis, and the release of proteases, cytokines, and ROS. While these responses are initially protective, clearing damaged cells and pathological deposits to maintain neural stability can become detrimental in chronic pathological states. In such conditions, prolonged activation leads to sustained phagocytosis, diffusion of pathological deposits, chronic neuroinflammation, and impaired phenotype transformation, thereby accelerating disease progression and neuronal degeneration.

Emerging evidence indicates that microglial metabolic dysfunction may act as an early trigger for ND, underscoring the significance of metabolic reprogramming and bioenergy regulation in maintaining neurological function. The dual role of microglia as both protectors and contributors to neurodegeneration underscores their transition from a protective to a pro-inflammatory or cytotoxic phenotype, which plays a critical role in the progression of disease.

Future research should prioritize the precise regulation of microglial functions by exploring the mechanisms of metabolism and phenotype transition. Efforts to enhance their protective phenotype, reduce neuroinflammation, and slow disease progression offer promising avenues for new therapeutic strategies in ND. Although the existing research has made substantial advancements in elucidating the role of microglia in cellular inflammation, how metabolic reprogramming influences microglial function, neuroinflammation, and brain health remains unknown. This is particularly important as many ND involve brain energy metabolism and inflammation disruptions.

This review emphasizes the interaction between metabolic reprogramming, microglial polarization, and their contributions to several ND. However, critical gaps remain. Most current insights into the metabolic regulation of microglia come from simplified *in vitro* models, such as cultured microglia, which lack the complexity of *in vivo* systems. Validation in sophisticated models, including the actual trigger of microglial metabolic breakdown in inflammatory diseases, is essential. Moreover, the causes of lipid metabolism dysregulation in microglia during ND remain poorly understood.

Research into the roles of metabolic reprogramming and inflammation in microglial biology is still in its infancy; deeper exploration is needed to clarify how these pathways interact within microglia and the broader brain environment. Therefore, understanding the integration of metabolism and polarization in microglia, along with strategies for regulating immune pathways, holds great promise for discovering new therapies for neuroinflammatory diseases.

## Data Availability

Bibliometric data statistics can be found on the Web of Science.
